# Influence of Post-Translational Modifications on Protein Identification in Database Searches

**DOI:** 10.1021/acsomega.0c05997

**Published:** 2021-03-15

**Authors:** Fanni Bugyi, Dániel Szabó, Győző Szabó, Ágnes Révész, Veronika F. S. Pape, Eszter Soltész-Katona, Eszter Tóth, Orsolya Kovács, Tamás Langó, Károly Vékey, László Drahos

**Affiliations:** † Institute of Organic Chemistry, Research Centre for Natural Sciences, Magyar Tudósok krt 2, H-1117 Budapest, Hungary; ‡ Hevesy György PhD School of Chemistry, Eötvös Loránd University, Pázmány Péter sétány 1/A, H-1117 Budapest, Hungary; § Faculty of Informatics, Eötvös Loránd University, Pázmány Péter sétány 1/C, H-1117 Budapest, Hungary; ∥ Department of Physiology, Faculty of Medicine, Semmelweis University, Tűzoltó utca 37-47, H-1094 Budapest, Hungary; ⊥ ELKH Supported Research Groups, Gellérthegy u. 30-32, H-1016 Budapest, Hungary; # Institute of Enzymology, Research Centre for Natural Sciences, Magyar Tudósok krt 2., H-1117 Budapest, Hungary; ∇ Department of Genetics, Cell- and Immunobiology, Semmelweis University, Nagyvárad tér 4, H-1089 Budapest, Hungary

## Abstract

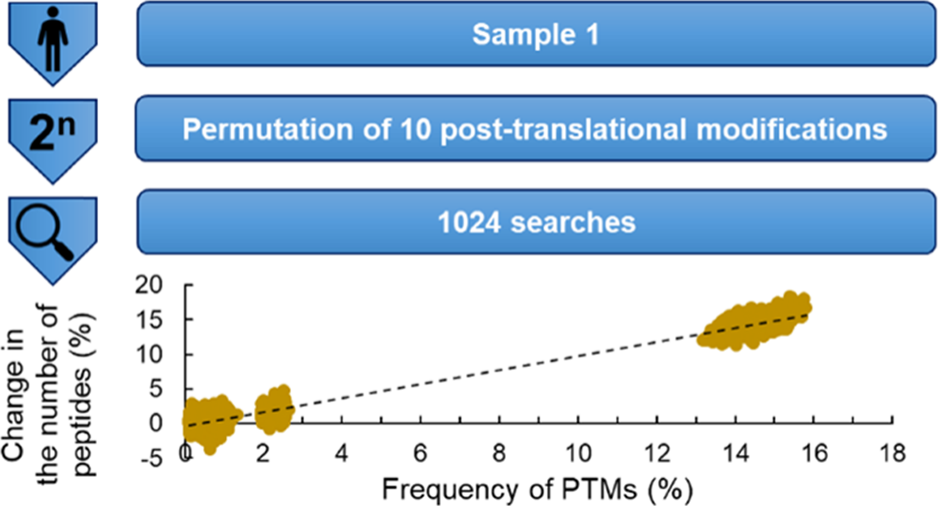

Comprehensive analysis of post-translation modifications (PTMs) is an important mission of proteomics. However, the consideration of PTMs increases the search space and may therefore impair the efficiency of protein identification. Using thousands of proteomic searches, we investigated the practical aspects of considering multiple PTMs in Byonic searches for the maximization of protein and peptide hits. The inclusion of all PTMs, which occur with at least 2% frequency in the sample, has an advantageous effect on protein and peptide identification. A linear relationship was established between the number of considered PTMs and the number of reliably identified peptides and proteins. Even though they handle multiple modifications less efficiently, the results of MASCOT (using the Percolator function) and Andromeda (the search engine included in MaxQuant) became comparable to those of Byonic, in the case of a few PTMs.

## Introduction

The identification and characterization of proteins and peptides is a key step in proteomics. Today, one of the most important and challenging tasks is the deeper understanding of the associations and interactions between the post-translational modifications (PTMs). This includes identifying, site localizing, and quantifying these modifications under different conditions (e.g., healthy and diseased organisms).^1−3^

The most common technique for the large-scale identification and characterization of proteins and peptides and their PTMs is tandem mass spectrometry via database search engines. The search engine generates all possible tandem mass (MS/MS) spectra of the peptides of proteins found in the database and compares these theoretical spectra with the experimental MS/MS spectra.^4^ The search algorithm assigns a score to each peptide spectrum match (PSM) corresponding to the quality of the hit. The best candidates, usually with the highest score, are accepted for identification.^5^ Some examples of database search engines are SEQUEST,^6^ MASCOT,^7^ MaxQuant,^8^ and X! Tandem.^9,10^

A proteomic search requires a large number of input parameters, which have an extremely large impact on the search results. Examples of these parameters include the mass accuracy, fragment ion types, number of missed cleavages, protease cleavage sites, taxonomy, sequence database, and the list of modifications.^11,12^ Incorrect setting of search parameters can yield results leading to erroneous biological conclusions.^13^ This is the consequence of their impact on the size and composition of the search space. By definition, the search space of a proteomics database search is “the collection of all possible peptide and fragment ions that need to be taken into account when a spectrum is searched. The number of possible peptides is the number of peptides that can be matched to a precursor *m*/*z* in the experimental data.”^14^ Even if all of the input parameters are the same, different search engines may generate unique search spaces.^15^

The list of included modifications is the parameter that probably requires the most careful consideration from a researcher, who is planning to perform a proteomic search. A modification can be treated in two ways, either being set as fixed or variable. A fixed modification does not increase the search space, as it only shifts the *m*/*z* value of candidates by the mass of the modification. In contrast, when considering a variable modification, all potential localization sites must be taken into account with all possible distributions of that modification. This leads to a steep increase in the size of the search space, which scales exponentially with the inclusion of additional modifications.^14^

Even though an extended search space broadens the range of discoverable peptides, the increased size can also have profound negative effects. One consequence of a larger search space is the longer search time as each of the candidates needs to be evaluated individually. Furthermore, it increases the probability of incorrect PSMs with a high score. This either leads to an increased number of false-positive identifications or raises the threshold above which identification is considered to be reliable, thus it may limit the number of identified compounds.^16,17^ The rate of false identifications is expressed by the false discovery rate (FDR), which increases in value for the same score threshold as the search space becomes larger.^18^ Accordingly, it may not be worthwhile to consider modifications in the searches. One of the most commonly used search engines, MASCOT, also suggests that if the goal is to identify as many proteins as possible, only a few modifications should be included in the search, or none at all.^19^

On the other hand, a significant portion of proteins carry post-translational modifications, and if these PTMs are not considered in the search, these components may remain unidentified. Chick et al. concluded that proteins with modifications are responsible for at least one-third of unassigned spectra.^20^ Based on these arguments, it would be worthwhile to include as many PTMs as possible in the search.

However, search engines utilizing classical database search algorithms (e.g., MASCOT) can struggle with the comprehensive analysis of PTMs. In the case of these engines, the more modifications are considered, the fewer spectra are identified at a given FDR. This phenomenon led to the development of novel search strategies that can identify as many known and, in cases, even unknown PTMs as possible in the sample.^21^ Multipass search strategies, like ISPTM^22^ or “cleaned search”^16^ approaches, can efficiently restrict the search space, using one basic search with no variable modifications and several iterative searches with a small number of considered modifications.^23^ The combination of de novo sequencing^24^ and database methods has resulted in the creation of various hybrid methods like Byonic,^25^ Open-pFind,^26^ and InsPecT.^27^ These hybrid search engines usually determine a partial sequence (two to four amino acids) of the experimental MS/MS spectrum by de novo sequencing and generate peptide candidates from the database for this sequence. This method significantly reduces the search space, thus reducing the time required for the search while the identification stays sensitive.^4^ Hybrid methods are excellent for analyzing hundreds of different modifications or even identifying unknown modifications.^28^ McClintock et al. searched for more than 40 oxidative mass shifts in a single search with InsPecT and Byonic software packages.^29^ Extensive PTM discovery can be performed in an unrestrictive way, although these open modification search tools require extremely long search times.^21^

In the present work, we study the effect of preselected PTMs, with the objective of giving practical advice on how to perform proteomic searches. We performed searches with three different search engines: Byonic hybrid search engine, which is well known for its efficiency when searching multiple modifications; MASCOT, which is one of the oldest classical database search engines; and MaxQuant, which is an extremely popular search engine for quantification. We discuss which modifications are worth considering in a proteomic search if the main objective is the identification of proteins and peptides. In addition, the huge difference between hybrid and database search engines, in terms of their treatment of multiple PTMs in a single search, is also presented.

## Results and Discussion

In a proteomic search, the post-translational modifications that are considered in the search significantly affect the efficiency of the identification, i.e., the number of identified peptides and proteins. We investigated when it is worth considering a modification in a search to maximize the number of reliably identified peptides and proteins. Across six search series, 2288 searches were performed by Byonic on five different samples. In total, 27 different PTMs were systematically studied. The correlation of the number and frequency of the considered PTMs to the number of identified components was studied in the case of multiple samples. The effect of an artificially increased search space was also investigated. Finally, we selected a few searches from the thousands of Byonic searches and performed them using classical database search engines, MASCOT and MaxQuant, to assess their usefulness in peptide discovery in the presence of multiple PTMs.

### Effect of the Number of Considered PTMs on the Number of Identified Components in Search-Series 1

We investigated the effect of the number of considered PTMs on search efficiency. We systematically considered every combination of 10 modifications present in sample 1 (Table 2); thus, 1024 searches were performed. Note that sample 1 was treated with the alkylation agent *N*-ethylmaleimide (NEM), which is the most frequent modification in the sample.

**Figure 1:**
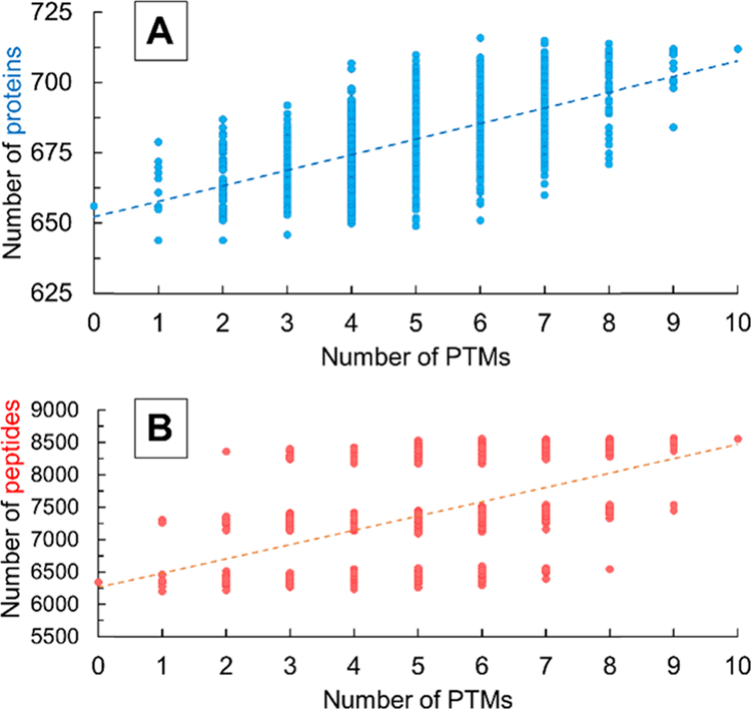
Relationship between the number of proteins (A) and peptides (B) reliably identified and the number of modifications considered in search-series 1.

Figure 1A shows the number of reliably identified proteins and Figure 1B shows the number of reliably identified peptides as a function of the number of considered PTMs. For example, out of 10 modifications, 4 modifications can be selected in 210 ways, and thus there are 210 dots in the figure when the number of considered PTMs is 4.

As Figure 1A shows, on average, the number of reliably identified proteins (see the Experimental Section for more details) increases with the number of considered modifications. This linear trend can be explained by the increasing number of identified peptides with PTMs. When no modification was considered, 656 proteins were reliably identified, while when all 10 modifications were considered, the number of reliably identified proteins increased to 712. Interestingly, the highest number of proteins (716) was identified using a specific combination of six modifications (namely, acetyl, carbamyl, crotonyl, amidation, NEM, and NEM hyd). It means some PTMs may increase, while others may decrease the number of identified components. This emphasizes the importance of the careful selection of the modifications that are to be included in the proteomic search.

A linear trend was also observed for the number of identified peptides (Figure 1B). An important difference compared to the case of the proteins is that the number of identified peptides can be isolated into three groups. The two most common modifications (NEM and NEM hyd, both having approximately 15% frequency) cause a significant growth in the number of identified peptides. If neither of these two modifications was considered, 6000–6500 peptides were identified; if only one of the two modifications was considered, 7000–7500 peptides were identified; and if both were considered, on average, 8500 peptides could be identified. These two modifications significantly facilitated the identification of peptides by their high frequency.

**Figure 2:**
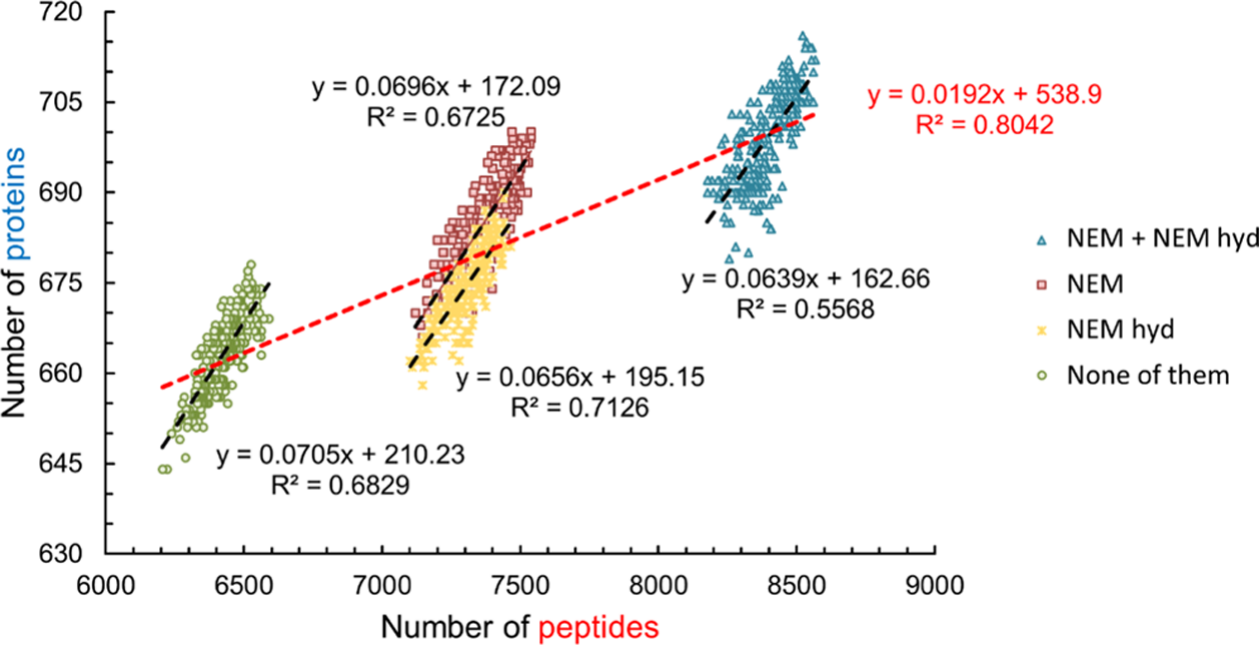
Correlation between the number of identified proteins and the number of identified peptides. Blue triangles represent the searches in which both NEM and NEM hyd modifications were considered, burgundy squares represent the searches in which NEM modification was considered, yellow Xs are those in which NEM hyd modification was considered, and green dots are those in which neither NEM nor NEM hyd modifications were considered. The black trend lines are fitted to these four groups separately, while the red trend line is fitted to all data points.

Considering more and more PTMs increases the number of identified peptides significantly. In the case of proteins, this effect is less pronounced (Figure 1), although there is a good correlation between the number of identified peptides and proteins (Figure 2). However, the sudden growth in the number of identified peptides caused by the two frequent modifications (NEM and NEM hyd) does not occur in the number of proteins. The overall trend line (red in Figure 2) is determined mainly by these two modifications. When combinations of the two frequent PTMs were considered separately (the four groups of points in Figure 2), it is possible to fit trend lines on these as well. These are shown by the black trend lines in Figure 2, and they are determined by the rare modifications. Considering even one of the most common PTMs increases the number of identified proteins significantly; however, the number of peptides increases at an even higher rate (the slope in the correlation plot of the number of peptides and proteins is 0.02). This relates to the identification of a large number of modified peptides of previously identified proteins. On the other hand, the inclusion of rare modifications does not have the same effect. In this case, the discovered peptides primarily belong to newly identified proteins (the slope of the black trend lines in Figure 2 is, on average, 0.07). When no common PTM was considered, on average, 9–10 identified peptides belonged to each of the identified proteins in proteomic searches (green dots in Figure 2). When one of the common modifications was considered, this increased to 11 peptides. When both modifications were considered, the number of identified peptides per protein increased to 12. This also means that, on average, the consideration of one common PTM resulted in the identification of ca. one modified peptide per protein.

So far, it can be stated that considering more modifications in a search increases the efficiency of the identification on average. Some PTMs significantly increase the number of identified peptides, while other PTMs may decrease that. We noted that this effect depends on the frequency of PTMs, so we explored the correlation between the frequency of individual modifications and the number of identified components below.

### Effect of the Artificially Increased Search Space

**Figure 3:**
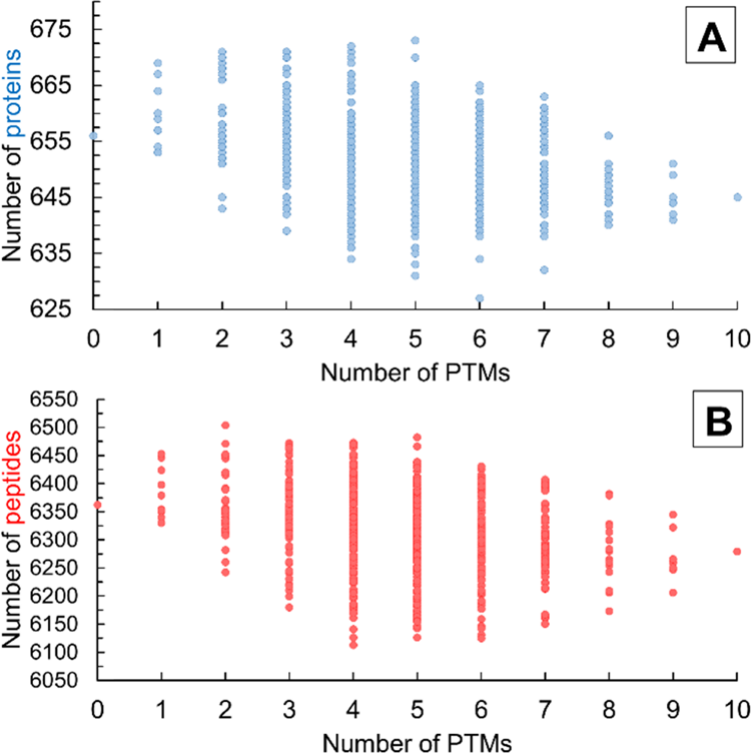
Relationship between the number of reliably identified proteins (A) and peptides (B) and the number of considered modifications in search-series 2, when an artificially increased search space was investigated.

We also investigated the effect of a growing search space on the number of identified components by considering several modifications that were not present in sample 1 (search-series 2). As more of these modifications were considered in the search, fewer proteins (Figure 3A) and peptides (Figure 3B) were reliably identified. Compared to the case when no modifications were included, only 200 peptides and 24 proteins were lost when 10 modifications were considered, which were not present in the sample. However, the inclusion of these 10 PTMs led to the identification of further 117 peptides and 13 proteins, which must be false-positive results. Therefore, at least in the case of Byonic, the increasing size of the search space hinders peptide and protein identification only to a small degree. This means that a relatively large number of PTMs can be considered without significant compromise.

### Effect of Frequency of PTMs

We found that the common modifications significantly increase the number of identified peptides (Figure 1B) and further investigated this correlation. At what level of frequency should a PTM be included in the search to increase the number of reliably identified peptides? The higher the frequency of the considered PTMs, the more peptides get identified. But will it always identify fewer peptides by considering PTMs with lower frequency?

**Figure 4:**
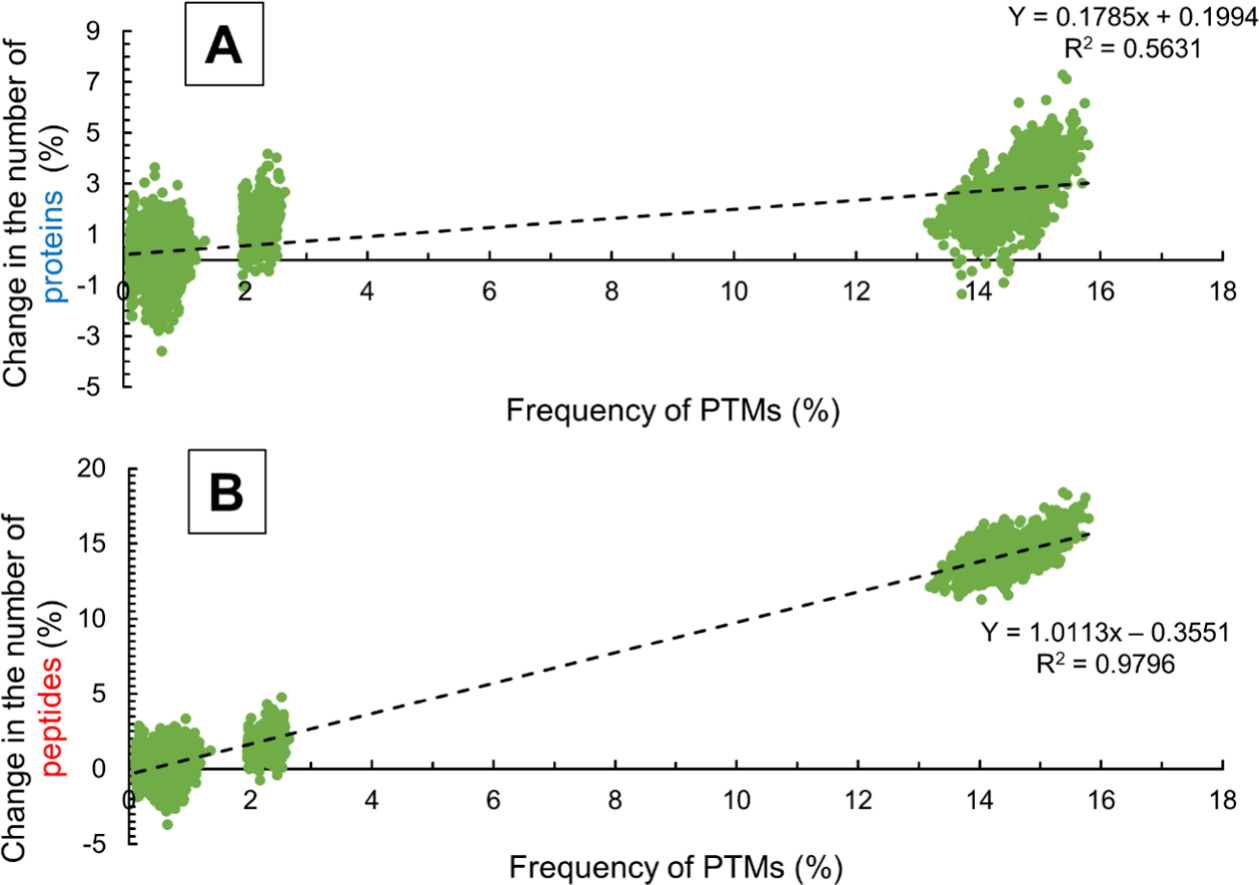
Dependence of the change in the number of reliably identified proteins (A) and peptides (B) on the frequency of the considered modification in search-series 1.

Figure 4B shows the effect of the frequency of modifications on the number of identified peptides. Each dot represents a search pair that consists of two searches between which the only difference is the consideration of one PTM. In the first search, the investigated PTM was included, while this modification was omitted in the second search, but all of the other parameters were the same, including other modifications. The number of peptides identified in the two searches was compared. For rare modifications (frequency < 1.5%), the number of peptide hits randomly increased or decreased within ±4% in the second search. Considering modifications with 2–2.5% frequency increases the number of identified peptides on average. Only a few cases were observed when the number of identified peptides was reduced by 0–1%. By considering modifications with ∼15% frequency, the number of reliably identified peptides increases significantly, with ∼15% on average.

The investigation of the change in the number of identified proteins (Figure 4A) uncovered a similar trend as the investigation of the change in the number of identified peptides. However, the number of protein hits increases more slowly compared to the increase presented in the case of the peptides. This is because proteins are identified using multiple peptides, and the identification of additional peptides does not necessarily lead to the identification of additional proteins.

**Figure 5:**
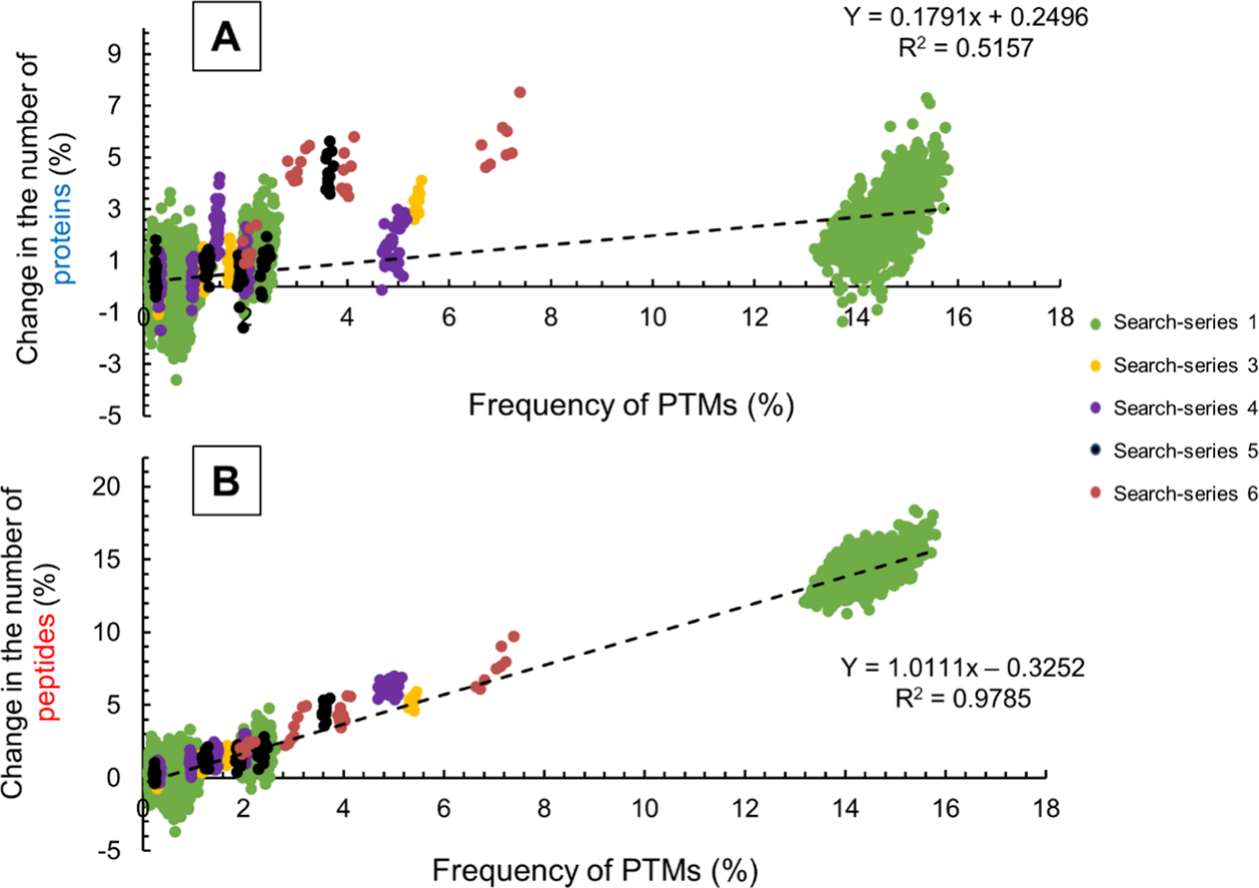
Relationship between the change in the number of reliably identified proteins (A) and peptides (B), and the frequency of the considered modifications in search-series 1 and 3–6.

The results presented so far originate from different evaluations (search-series 1 and 2) of sample 1. To evaluate whether the observed trends are general or specific only for this sample, we investigated four additional biological samples with their related PTMs included in the respective searches (see Table 2). The combined results of the evaluation of the five samples are shown in Figure 5. In the case of these further samples, the number of modifications that were included in the permutation varied between 4 and 10; therefore, the numbers of searches also differed among the individual search series. In Figure 5, each search series is marked by a unique color.

As can be seen in Figure 5B, the relationship between the number of identified peptides and the frequency of the modifications is nearly linear within the studied range. Modifications increase the number of peptide hits according to their frequency; however, modifications with low frequency can also impair the efficiency of identification. In accordance with the literature,^16^ it is important to consider a variable modification if it is abundant. Based on our results, it can be stated that it is worth considering modifications with over 2% frequency in searches.

The change in the number of identified proteins (Figure 5A) also yielded a similar curve as it was in the case of search-series 1. However, in the case of certain samples, the trend of identifying new proteins by including PTMs with frequency >2% is even more favorable (e.g., search-series 5 and 6).

### Effect of the Frequency of PTMs Using MASCOT and MaxQuant

**Figure 6:**
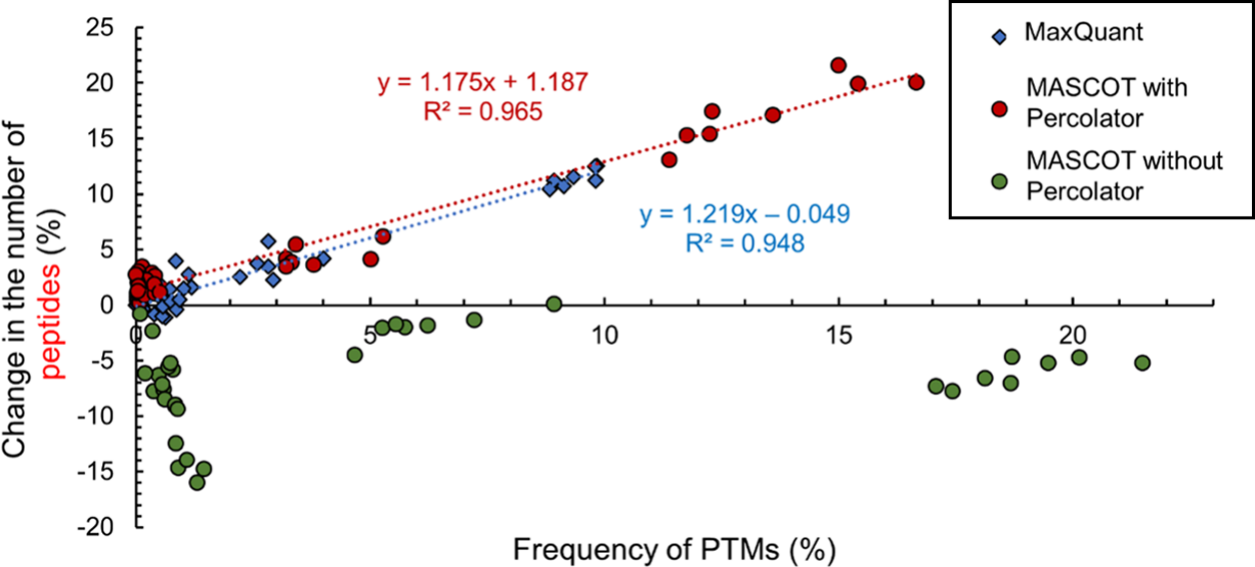
Dependence of the change in the number of reliably identified peptides by MASCOT (for identification, Percolator function was (red dots) and was not (green dots) used) and MaxQuant (blue dots) database search engines on the frequency of the considered modifications.

For comparison, a couple of searches from search-series 1 were also performed with MASCOT and MaxQuant, which are two of the most frequently used software in proteomics. For technical reasons (individual parameter files need to be created manually), only 21 searches were performed with both of the search engines. Based on these experiments, clear tendencies and differences can be observed (Figure 6).

The consideration of PTMs in the MASCOT searches (green dots in Figure 6) clearly has a negative influence on the number of reliably identified peptides, regardless of the frequency of these modifications. In these searches, only up to three modifications were considered. In all searches, when a PTM was considered, fewer peptides were identified than in searches when no modification was considered. A single modification could reduce the number of identified peptides by even 16% due to the explosion of the search space. The larger the search space is, the higher the identification thresholds are, and it becomes less likely to identify a component.

Searches were also performed with the use of Percolator (red dots in Figure 6). Percolator is an algorithm in MASCOT that uses semisupervised machine learning to improve the distinction between correct and incorrect spectral matching, resulting in a more reliable list of protein hits.^30^ The Percolator significantly improves the peptide identification, resulting in a trend that is similar to the results achieved with Byonic (shown in Figure 5). Interestingly, Percolator achieves this without directly influencing the search space itself.

Andromeda search engine was also investigated with MaxQuant (blue dots in Figure 6). It suggests that the relationship between the change in the number of identified peptides and the frequency of the considered PTMs is also similar to this trend of Byonic. However, the frequencies of the most frequent modifications (approximately, 15% in Byonic searches) are about 10%. It means that in sample 1 peptides with NEM or NEM hyd, modifications were identified at a lower rate. In this case, the number of all identified peptides was also lower than in the searches performed with Byonic.

Even though a linear relationship could not be observed between the number of identified proteins and the frequency of modifications, we found that the inclusion of PTMs with at least 1.5–2% frequency significantly improved the efficiency of protein identification (data not shown). The consideration of these PTMs increases the number of protein hits by 1–8%, while the consideration of PTMs with <1.5–2% frequency decreases the protein hits in all three cases.

In general, it is not advisable to use classical database search engines for the comprehensive study of PTMs. In MASCOT searches, the use of Percolator is essential when this kind of search engine is applied. However, only a small number of PTMs can be investigated because even with the use of Percolator, when more than three modifications were included in a search, fewer peptides were identified (see the Supporting Information Figure S1). In the case of MASCOT without Percolator, considering each additional PTM (starting with the first) leads to a further decrease in the number of identified peptides. MaxQuant results show similarities to those of MASCOT with Percolator; however, the inclusion of additional modifications increases the search time drastically. The inclusion of two additional PTMs in a search (4 instead of 2) nearly doubled the required time for the search, increasing it from 3 to 4 h, depending on the particular modifications, to 5–8 h.

## Conclusions

In the case of unknown samples, considering PTMs is an iterative procedure. First, one has to determine which PTMs are present in the sample (for example, using so-called “open searches”^21^). Second, determining (e.g., based on the number of identified peptides) the approximate frequency of the PTMs in the sample (or in a series of samples). Last, based on the results of this paper, one can decide which PTMs should be included in the proteomic analysis.

We used several thousand proteomic searches to systematically investigate the effect of the number of considered PTMs in database search. Increasing the number of PTMs had very little drawback on Byonic searches; considering 10 PTMs, which were not present in the sample, decreased the number of protein hits only by ∼2%. A linear relationship was found between the frequency of the PTM occurring in the sample and the number of components identified. For example, considering a PTM with ∼15% frequency increases the number of peptide hits by about 15% and protein hits by ∼3%. Modifications with over 2% frequency are worth considering, as these will increase peptide and protein identifications. We found that MASCOT was not capable of dealing with the increased search space required for considering multiple PTMs. However, using the Percolator function of MASCOT (based on a machine learning algorithm) improved the results significantly. In the case of only a few PTMs, using MASCOT with Percolator or MaxQuant leads to results that are comparable to that of Byonic.

## Experimental Section

Protein and PTM identifications were performed using Byonic (v3.6.0, Protein Metrics Inc.), MASCOT (v2.5, Matrix Science, England), and MaxQuant (1.6.17.0, Max-Planck-Institute of Biochemistry, Germany) search engines. Due to a large number of database searches, the creation of Byonic parameter files and the evaluation of the search results were performed with the help of in-house scripts.

### Byonic Searches

Systematic investigation of the effect of PTMs added to the list of modifications was achieved by evaluating the performance of six search series. Within a search series, we individually included and omitted every PTM on the list, while the rest of the parameters, including other PTMs, were kept the same. Therefore, the investigation of 10 modifications meant 1024 possible PTM combinations and 1024 searches. The search series were performed on tryptic digests of five different human cell lines. The analysis of the samples was previously performed in the research group with a nanoLC-MS/MS setup using a Dionex Ultimate 3000 RSLC nanoLC (Dionex, Sunnyvale, CA) coupled to a Bruker Maxis II Q-TOF (Bruker Daltonik GmbH, Bremen, Germany) via a CaptiveSpray nanoBooster ionization source. Peptides were separated on a 1.7 μm, 75 μm × 250 mm Acquity M-Class BEH130 C_18_ analytical column (Waters, Milford, MA) using a 90 min linear gradient elution. MS and MS/MS spectra were recorded with a cycle time of 2.5 s in the range of 150–2200 Th *m*/*z*. MS spectra were recorded at 3 Hz. After CID fragmentation, ions from high-intensity precursors were recorded at 16 Hz, while ions from low-intensity precursors were recorded at 4 Hz. Detailed information about the samples and the list of the considered modifications in their respective searches can be found in Table 1. The placenta sample and linked clinical data were collected at the Maternity Clinic (Budapest, Hungary). The specimen and data were stored anonymously in the Perinatal Biobank of the Research Centre for Natural Sciences in Budapest. Sample and data collection was approved by the Health Science Board of Hungary (ETT-TUKEB 4834-0/2011-1018EKU), and the study was performed based on the principles of the World Medical Association Declaration of Helsinki. Written informed consent was obtained from the patient before sample collection.

**Table 1:** Sample Information and Their Modifications

sample	sample information	PTMs identified
sample 1	human skin (HaCaT cell line)	see Table 2
sample 2	human embryonic kidney (HEK 293 cell line)	carbamidomethyl; oxidation; Gln → pyro-Glu; Glu → pyro-Glu; ammonia-loss; acetyl
sample 3	human bone tissue	carbamidomethyl; oxidation; Gln → pyro-Glu; Glu → pyro-Glu; ammonia-loss; acetyl
sample 4	human placenta tissue	carbamidomethyl; oxidation; Gln → pyro-Glu; Glu → pyro-Glu; ammonia-loss; acetyl; Delta:H(2)C(2)
sample 5	leukemia (HL60 cell line)	carbamidomethyl; dioxidation; deamidated; Gln → pyro-Glu; Glu → pyro-Glu; ammonia-loss; acetyl; +59.019355; +116.040819

The selection of the particular PTMs for the investigation of the effects of modifications on proteomic searches was based on the result of Byonic Preview. Preview is a companion tool for Byonic, which can evaluate the optimal settings for the search in advance.^31^ In the case of sample 1, Byonic Wildcard search was also performed for finding sample preparation-related PTMs. A Wildcard search enables us to identify residues (unanticipated modifications) within a user-settable mass delta range.^32^

Search-series 1 was performed on sample 1, using a focused database for limiting the search space. This focused database contained 1435 proteins, which were identified in a preliminary database search performed on a complete human database (parameters used for this search can be found in Table S1 in the Supporting Information). The mass accuracy was set to 10 ppm for the precursor ions and 20 ppm in the case of fragment ions. Cleavage sites were set at the C-terminus of lysine (K) and arginine (R), with a maximum of two missed cleavage sites being permitted. Carbamidomethylation on cysteine was set as a fixed modification. The list of variable modifications considered in the search and their individual frequencies are shown in Table 2. The frequency of PTMs was calculated in the following way: in a search in which a given PTM was considered, the number of peptides identified with the PTM was divided by the total number of peptides identified in that search. Then, the ratios of the individual searches were averaged across all searches. *N*-ethylmaleimidation (NEM) and its hydrolysis product (NEM hyd) are the most common modifications, both having approximately 15% frequency. This is because the sample was prepared using the NEM chemical modification. For protein identification, 1% FDR was allowed.

**Table 2:** List of Variable PTMs Considered in Search-Series 1

name	mass difference	position	frequency of PTMs (%)
dicarbamidomethyl	+114.042927	N-term	0.71
acetyl	+42.010565	N-Term, Protein N-Term	2.25
Glu → pyro-Glu	–18.010565	N-term E	0.15
carbamyl	+43.005814	N-term, K	0.67
oxidation	+15.994915	M	0.64
crotonyl	+68.026215	K	0.67
amidation	–0.984016	C-term	0.72
hydroxymethylOP	+108.021129	K	0.17
*N*-ethylmaleimide (NEM)	+125.04767	N-term, K, N, C	14.97
*N*-ethylmaleimide hydrolysis (NEM hyd)	+143.058243	N-term, K, H, C	14.06

The effect of 10 modifications was investigated by performing 1024 Byonic searches. In all cases, a probability of false protein identification was 1% or less (AbsLogProb value ≥ 2) and at least two unique peptides were needed for a protein to be accepted as “reliably identified”. The criterion for the acceptance of peptides was less than 5% probability of false identification (AbsLogProb ≥ 1.3). Note that while the AbsLogProb values include the effect of search space size, they are based on Byonic’s own probabilistic model instead of decoy proteins and so the percentages cannot be claimed to be proper FDR values.

In search-series 2, we artificially increased the search space by considering modifications that were not present in the sample (e.g., chemical isotope labeling) to test the capability of the Byonic search engine to control the growth of the search space. The search parameters, except for the PTM list, were the same as in search-series 1. The list of PTMs used in this search series can be found in Table 3.

**Table 3:** List of PTMs Considered in Search-Series 2

name	mass difference	position
delta:H(2)C(3)O(1)	+54.010565	K, R
piperidine	+68.062600	N-Term, K
methylphosphonate	+77.987066	S, T, Y
Trp → oxolactone	+13.979265	W
label:13C(5)15N(1)	+6.013809	E, M, P, V
label:13C(6)15N(1)	+7.017164	I, L
label:13C(6) + acetyl	+48.030694	K
label:13C(6) + dimethyl	+34.051429	K
label:13C(6)15N(4) + methyl	+24.023919	R
label:13C(9)	+9.030193	F, Y

Search-series 3–6: The methodology described for search-series 1 was repeated on samples 2–5 with their relevant modifications (see Table 1) to further consolidate the conclusions drawn from the results of search-series 1. Detailed parameters of these search series are shown in Table S2 in the Supporting Information.

### MASCOT and MaxQuant Searches

We also investigated the operation of purely database search engines, MASCOT and Andromeda (using MaxQuant), by selecting 21 searches taken from search-series 1. These examples were chosen arbitrarily but with regard to them being search pairs (two searches between which one PTM was the only difference). Up to three of the variable modifications listed in Table 2were considered in these searches (for the modification lists of these individual searches, see the Supporting Information Table S3).

The type of MASCOT search was MS/MS Ion Search. The searches were performed on a complete human database (20244 sequences). The mass accuracy of precursor ions was set to 10 ppm and the mass accuracy of fragment ions was set to 0.02 Da. Cleavage sites were set at the C-terminus of lysine (K) and arginine (R), with a maximum of one missed cleavage. Carbamidomethylation (C) was set as a fixed modification. Data processing was performed with and without Percolator.

The parameters of MaxQuant searches were as similar to the MASCOT settings as possible; the searches were performed on the focused database of search-series 1.

## Supporting Information Available

The correlation between the number of considered modifications and the change in the number of identified peptides in the case of database searches (Figure S1), search parameters for creating the focused database for search-series 1 (Table S1), search parameters for search-series 1–6 (Table S2), and the list of MASCOT and MaxQuant searches (Table S3) (XLSX)

## Notes

Data sets (for search-series 1–3 and 6) can be accessed via the MassIVE repository (MSV000086753).

## Abbreviations

FDR: false discovery rate
MS: mass spectrometry
NEM: *N*-ethylmaleimide
NEM hyd: *N*-ethylmaleimide hydrolysis
PTM: post-translational modification
PSM: peptide spectrum match
